# A Digital Mental Health App Incorporating Wearable Biosensing for Teachers of Children on the Autism Spectrum to Support Emotion Regulation: Protocol for a Pilot Randomized Controlled Trial

**DOI:** 10.2196/45852

**Published:** 2023-06-26

**Authors:** Emma H Palermo, Amanda V Young, Sky Deswert, Alyssa Brown, Miranda Goldberg, Evan Sultanik, Jessica Tan, Carla A Mazefsky, Lauren Brookman-Frazee, James C McPartland, Matthew S Goodwin, Jeffrey Pennington, Steven C Marcus, Rinad S Beidas, David S Mandell, Heather J Nuske

**Affiliations:** 1 Penn Center for Mental Health Perelman School of Medicine University of Pennsylvania Philadelphia, PA United States; 2 Alevio LLC Philadelphia, PA United States; 3 School of Dental Medicine University of Pennsylvania Philadelphia, PA United States; 4 Department of Psychiatry University of Pittsburgh Pittsburgh, PA United States; 5 Department of Psychiatry University of California San Diego San Diego, CA United States; 6 Yale Child Study Center Yale University New Haven, CT United States; 7 Bouvé College of Health Sciences Northeastern University Boston, MA United States; 8 Children's Hospital of Philadelphia Research Institute Children's Hospital of Philadelphia Philadelphia, PA United States; 9 School of Social Policy and Practice University of Pennsylvania Philadelphia, PA United States; 10 Department of Medical Social Sciences, Feinberg School of Medicine Northwestern University Chicago, IL United States

**Keywords:** digital mental health, just-in-time adaptive intervention augmentation, JITAI, autism, heart rate tracking, emotion dysregulation, challenging behavior, evidence-based strategies, student progress monitoring, mobile phone

## Abstract

**Background:**

As much as 80% of children on the autism spectrum exhibit challenging behaviors (ie, behaviors dangerous to the self or others, behaviors that interfere with learning and development, and behaviors that interfere with socialization) that can have a devastating impact on personal and family well-being, contribute to teacher burnout, and even require hospitalization. Evidence-based practices to reduce these behaviors emphasize identifying triggers (events or antecedents that lead to challenging behaviors); however, parents and teachers often report that challenging behaviors surface with little warning. Exciting recent advances in biometric sensing and mobile computing technology allow the measurement of momentary emotion dysregulation using physiological indexes.

**Objective:**

We present the framework and protocol for a pilot trial that will test a mobile digital mental health app, the *KeepCalm* app. School-based approaches to managing challenging behaviors in children on the autism spectrum are limited by 3 key factors: children on the autism spectrum often have difficulties in communicating their emotions; it is challenging to implement evidence-based, personalized strategies for individual children in group settings; and it is difficult for teachers to track which strategies are successful for each child. KeepCalm aims to address those barriers by communicating children’s stress to their teachers using physiological signaling (emotion dysregulation detection), supporting the implementation of emotion regulation strategies via smartphone pop-up notifications of top strategies for each child according to their behavior (emotion regulation strategy implementation), and easing the task of tracking outcomes by providing the child’s educational team with a tool to track the most effective emotion regulation strategies for that child based on physiological stress reduction data (emotion regulation strategy evaluation).

**Methods:**

We will test KeepCalm with 20 educational teams of students on the autism spectrum with challenging behaviors (no exclusion based on IQ or speaking ability) in a pilot randomized waitlist-controlled field trial over a 3-month period. We will examine the usability, acceptability, feasibility, and appropriateness of KeepCalm as primary outcomes. Secondary preliminary efficacy outcomes include clinical decision support success, false positives or false negatives of stress alerts, and the reduction of challenging behaviors and emotion dysregulation. We will also examine technical outcomes, including the number of artifacts and the proportion of time children are engaged in high physical movement based on accelerometry data; test the feasibility of our recruitment strategies; and test the response rate and sensitivity to change of our measures, in preparation for a future fully powered large-scale randomized controlled trial.

**Results:**

The pilot trial will begin by September 2023.

**Conclusions:**

Results will provide key data about important aspects of implementing KeepCalm in preschools and elementary schools and will provide preliminary data about its efficacy to reduce challenging behaviors and support emotion regulation in children on the autism spectrum.

**Trial Registration:**

ClinicalTrials.gov NCT05277194; https://www.clinicaltrials.gov/ct2/show/NCT05277194

**International Registered Report Identifier (IRRID):**

PRR1-10.2196/45852

## Introduction

### Background

Children on the autism spectrum can exhibit challenging behaviors, such as aggression, self-injury, and property destruction [1-3], which occur in as much as 80% of children [4]. These behaviors can have a devastating impact, negatively affect personal and family well-being [5], contribute to teacher burnout [6], and even lead to hospitalization [7]. Challenging behaviors may stem from a culmination of physiological and psychosocial stress when children are unable to regulate their stress, that is, when they do not have a repertoire of emotion regulation strategies to call on to defuse their stress [8,9]. Emotion dysregulation—difficulties with monitoring, evaluating, and modifying emotional responses to environmental demands [10]—is thought to serve as a mediator between stress and challenging behaviors in autism [11,12]. Consistent with this notion, recent literature has documented that challenging behaviors in children and adolescents with autism spectrum disorder are more likely to surface in response to situations placing demands that exceed their emotion regulation skills [13]. Individuals on the autism spectrum are more likely to experience stressful events compared with their peers owing to their difficulties with communicating, understanding others’ behaviors, and responding to demands; sensitivities to sensory stimuli; insistence on sameness; and social expectations that might be perceived as emotionally overwhelming [9]. These difficulties or factors can be particularly pronounced in the school setting [14]. Therefore, these difficulties with emotion dysregulation, in addition to frequently experienced stressors, place children on the autism spectrum at high risk for frequent challenging behavior episodes.

Evidence-based strategies for managing challenging behaviors and supporting emotion regulation in schools include proactive (antecedent) and reactive (consequent) strategies [1,15,16]. Proactive strategies, such as setting routines and tailoring the environmental or instructional design to prevent challenging behaviors, are typically preferred to reactive strategies, such as punishment and planned ignoring [1,15,16]. However, even when proactive strategies are well implemented, challenging behaviors still occur, with parents and teachers often reporting that they occur without warning [11,12]. In children on the autism spectrum, triggers can be difficult to determine even with expertise in functional behavioral assessment (which is not standard training for the teaching force) [17], as children on the autism spectrum often have trouble in communicating their stress [18]. Therefore, it is critical to understand the stress-related triggers that lead to the onset of challenging behaviors. Early detection of rising stress can allow for the implementation of proactive strategies targeting stress reduction or emotion regulation, such as emotion regulation training; mindfulness-based strategies; the prompting of cognitive reappraisal; functional communication training; or other antecedent-based strategies, such as providing access to sensory stimuli or offering choices.

A way to know when a child may be experiencing stress before a challenging behavior, and thus intervene early, is by measuring the physiological state; physiological state is a well-recognized index of stress, which may be captured using many physiological channels, including heart rate (HR) [19-24]. Many theoretical frameworks point to a causal pathway between physiological stress and challenging behavior [8,25-27], and previous studies have shown elevated HR before the onset of a challenging behavior in adults on the autism spectrum [28,29]. In our previous study, we found that there is often a >22% increase in HR in the 58 seconds immediately preceding the challenging behavior and that this increase has moderate utility in predicting challenging behaviors (area under the curve [AUC]=0.72; *P*<.001) [30]. We replicated this finding in 2 samples of children aged 8 to 12 years on the autism spectrum, finding that 33% to 36% increase in HR in the preceding 76 to 80 seconds was associated with challenging behaviors (AUC=0.75-0.82). These results were again replicated in another data set including both children and adolescents on the autism spectrum (AUC=0.71) [31]. These studies suggest that responding to early HR increases may be a more direct approach to identifying the need for emotion regulation strategies, which may support the identification of behavioral triggers.

Timely identification of stress may help prevent emotion dysregulation and challenging behaviors. In addition, children on the autism spectrum may be less equipped to deal with this stress, as many have well-documented difficulties in independently using emotion regulation strategies and instead more frequently rely on maladaptive or ineffective strategies, such as rumination or shutting down [32-37]. Thus, difficulty in emotion regulation is likely a mechanism by which stress contributes to challenging behaviors, and teaching emotion regulation is critical. However, there are 3 barriers that limit the use of emotion regulation strategies for managing challenging behaviors in children on the autism spectrum in school settings. First, teachers may not detect children’s stress-related triggers or their rising stress, with or without training, as children on the autism spectrum often do not express emotions verbally or in clear, observable ways using nonverbal communication channels [18]. Therefore, the detection of stress by teacher’s observation alone may be sometimes ineffective or unreliable.

Second, teachers work in dynamic and fast-paced classroom environments. Even when teachers are sensitive to behavioral triggers and evidence-based behavior plans are in place, teachers have multiple demands on their attention and may not recognize a trigger or recall the best strategies to use with each student in the moment of crisis. In addition, teachers’ own stress during episodes may interfere with this recall, as their stress has been found to interfere with delivery of evidence-based practices in schools [38,39]. Therefore, teachers need support to implement emotion regulation interventions with children on the autism spectrum.

**Figure 1:**
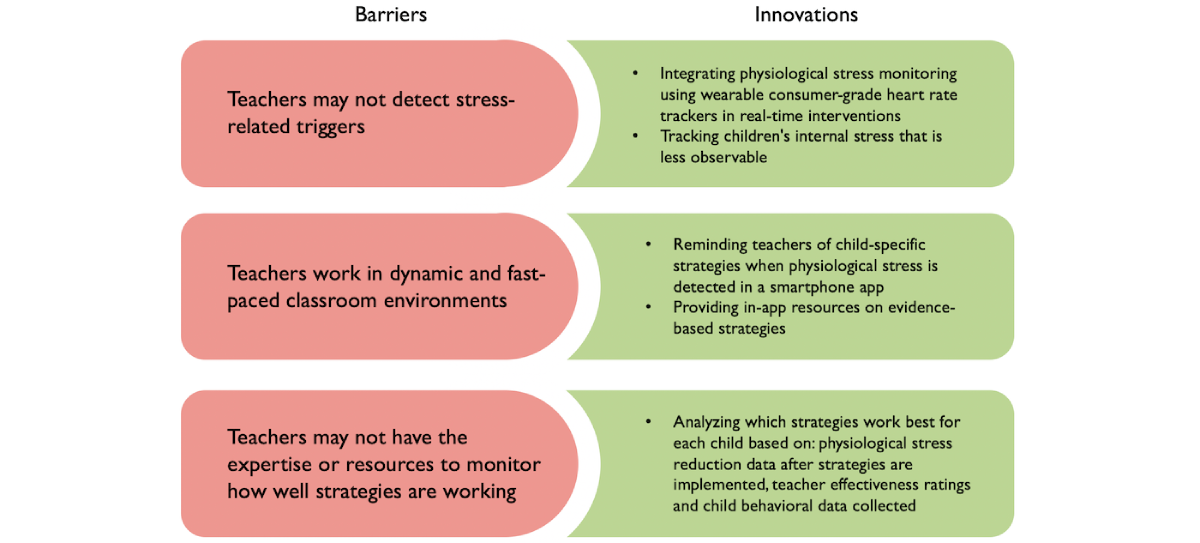
Barriers and the KeepCalm app’s corresponding proposed innovations to be tested.

Third, teachers may not have the training or resources to monitor how well the strategies are working. Many teachers do not receive training in program monitoring and analysis that is required to refine children’s behavioral plans, limiting their ability to make data-informed decisions about which strategies work for a given child. Even when teachers have training in outcome measurement and monitoring, they may be unable to systematically monitor the success of strategies owing to competing demands. They may not have the time, personnel, or technological resources to determine which strategies are the most effective. Our proposed mobile health (mHealth) app, KeepCalm, is designed to address these 3 barriers and reduce or prevent challenging behaviors in children on the autism spectrum (Figure 1; app development and features are described in the *Methods* section).

The KeepCalm app is designed as a real-time electronic clinical decision support tool, otherwise known as a *just-in-time adaptive intervention* (JITAI) augmentation, a term coined in mHealth [40]. JITAI augmentations are intervention supports designed to provide the right type or amount of support at the right time by adapting to a person’s changing internal and contextual state (eg, by tracking physiological stress, sleep quality, exercise or activity, temperature, and location). Most JITAI technologies are designed to support the individual client directly (eg, check-in messages and resources for clients with alcohol use disorder, triggered by GPS data showing that they are near a predetermined risk location, such as a local bar) [41]. However, the KeepCalm app is one of the first (also refer to the studies by Juarascio et al [42] and Clausen et al [43]) to support the provider (in this case, teacher or aide) directly, so that they may in turn best support their client (in this case, student).

### Objectives

We present the framework and protocol for a pilot randomized waitlist-controlled field trial that aims to examine the implementation of a digital mental health app, the KeepCalm app (refer to the *Materials* section for details about the app), designed for children on the autism spectrum with challenging behaviors of any IQ or speaking ability. Given that >95% of digital mental health apps have not actually been studied [44,45], an overarching goal of the project is to test the KeepCalm app systematically. We expect the KeepCalm app to score high on usability, acceptability, feasibility, and appropriateness. We also predict satisfactory proximal efficacy (>80% clinical decision support success rates and <20% false positive and negative rates), high technical reliability (the removal of artifacts and offline mode activation success), and the use of the KeepCalm app to result in low severity and frequency of challenging behaviors and emotional dysregulation in children on the autism spectrum after the trial relative to baseline.

## Methods

### App Development Work

We have used user-centered design [46,47] methods to develop the mHealth KeepCalm app by involving key stakeholders in each phase of development and testing [47-49]. As part of the early stages of the project, we have already conducted needs assessments with school personnel and parents to understand their needs in managing children’s challenging behaviors and supporting their emotional regulation. We are in the process of completing 5 cycles of rapid cycle prototyping (so far, 4 cycles have been completed, including 2 with the live app prototype). App feedback gathered through this testing has directly informed future app iterations. We have also gathered data showing high usability, feasibility, acceptability, and appropriateness of the KeepCalm app and data from associated HR trackers and accelerometry devices to examine in the context of regulatory and ethical educational frameworks [50]. Alongside this work, we conduct expert advisory board meetings with a board composed of relevant experts in the community (the autism support coordinator from the School District of Philadelphia, teachers, teacher coaches, parents, and adults with autism). The board meets every few months to gather their feedback about and support for every aspect of the project, including app features and design, in-app evidence-based strategy resources, study methods, and recruitment. All previous work will inform the final KeepCalm app to be tested in this pilot randomized waitlist-controlled field trial.

### Design

We will conduct a pilot randomized waitlist-controlled field trial of the KeepCalm app (refer to the *Materials* section for details about the KeepCalm app) in which participants will either be randomized to the KeepCalm app for a period of 3 months or will be included in a waitlist and will trial the app for 3 months after an initial 3-month waiting period. In this trial, the KeepCalm app will be tested for usability, feasibility, acceptability, and appropriateness, and we will also collect some preliminary proximal and distal efficacy data to inform a future, fully powered randomized field trial.

### Participants and Recruitment

Overall, 20 educational teams comprising a preschool or elementary school student on the autism spectrum, their parent, their teacher, and their aide (if they have one) will be recruited. If a child does not have an aide or their aide declines to participate, their speech therapist, occupational therapist, or another educational team member may participate instead. Therefore, at a minimum, teacher-child or parent dyads will be consented, with some teams forming a triad with an aide or other member of the educational team. Teachers may participate with 1 to 2 of their students on the autism spectrum.

### Inclusion Criteria

Children must have an educational classification of autism, verified using the Social Communication Questionnaire [51], a parent-reported measure. They must also exhibit challenging behaviors (ie, behaviors dangerous to the self or others, behaviors that interfere with learning and development, and behaviors that interfere with socialization). Examples include aggression, self-injury, escape behavior, property destruction, loud noises, and rigid or inflexible behavior (if at least one of the challenging behavior criteria mentioned previously applies).

Participants will be recruited from the School District of Philadelphia and private preschools or schools in the Philadelphia area using several recruitment methods including emailing teacher listserves or email lists, through the center participant registry, through the study’s expert advisory board, and through projects led by HJN or DM. We will also send Parent Permission-to-Contact forms home from school via students, along with the study flyer. If parents sign the form and send it back to school, we will reach out to them to invite them into the study. We also include a version of the flyer and Parent Permission-to-Contact form in Spanish. We may also use study flyers sent through various relevant organizations and listserves, newsletters, internet postings, social media (Facebook and Twitter), and other recruitment tools as needed, and we may attend parent workshops and autism-related events to recruit participants. If study participation is initiated by parents, after consenting parents, we will send the referral contact forms to schools via parents to recruit the child’s teachers or reach out to the schools directly to invite teachers to participate, after obtaining letters of support from school principals or directors, if required. Then, parents and teachers who agree to be contacted will be contacted by the research team to explain the study and invite them to participate. In addition, once teacher consent is completed, classroom aides will also be invited to participate. If no aides are available to participate, we will invite their speech therapist, occupational therapist, or any other educational team member identified by the parent or teacher.

To help characterize the children in the study, we will obtain the child’s IQ from their Individual Education Program, if this was derived from a standardized test battery. If such an IQ score is not available on the child’s Individual Education Program, the research team will administer the Stanford Binet Abbreviated Scales [52] test battery to obtain an IQ score.

### Materials

#### Overview of the KeepCalm App

**Figure 2:**
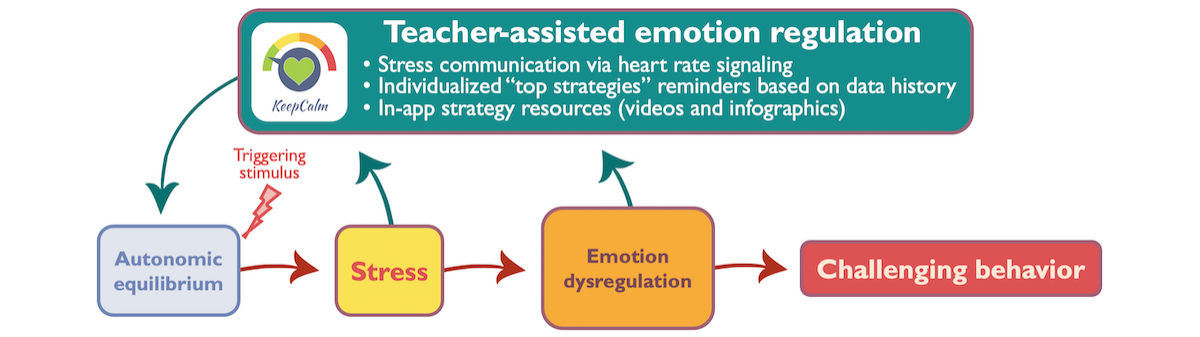
KeepCalm causal model.

The KeepCalm app is designed to be an interactive learning and data collection tool to facilitate a team approach to support emotion regulation in students. It integrates wearable HR tracking to identify and alert educators about heightened stress in their students and remind them about evidence-based strategies for de-escalating stress, supporting proactive coping, and preventing challenging behaviors. Refer to Figure 2 for the KeepCalm causal model, which shows that the pathway from stress to challenging behaviors via emotion dysregulation is “rescued” with the KeepCalm app, via teacher-assisted emotion regulation, whereby the teacher supports their student to reattain autonomic equilibrium (ie, physiological baseline or resting state) via stress communication (ie, child HR signaling to teacher), along with individualized recommendations about strategies to keep calm.

The KeepCalm app offers 3 innovations (to be tested) that address the 3 barriers described in the *Introduction* section (Figure 1). First, it incorporates physiological stress data into treatment delivery by capitalizing on recent advances in wearable biosensing [22,53,54]. The selected consumer-grade trackers are accurate [53,55,56] and have high technical reliability [55,57] for measuring HR (beats per minute) and HR variability (interbeat interval), making them inexpensive and scalable alternatives to medical-grade devices. In previous studies, we found that 85% of children on the autism spectrum will wear these trackers without complaint and rate them high on comfort and low on exacerbating sensory sensitivities [55,57].

Second, KeepCalm reminds teachers about child-specific strategies when physiological stress is detected. Computerized clinical decision support systems have been found to be related to higher fidelity in evidence-based practices and better patient outcomes among providers who work in chaotic environments, such as ambulatory clinic staff [58-61]. Despite the promise of this technology, such systems have not been linked with HR monitoring for decision support during behavioral interventions or therapy. The KeepCalm app gives pop-up notifications about top emotion regulation and behavior management strategies identified for each child and contains a comprehensive easy-to-access list of proactive and reactive strategies to support teacher training and ongoing in-class coaching in these strategies by the center’s teacher coaches. Additional coaching will be conducted as needed.

Third, it has a feature to track and tailor strategy recommendations based on stress reduction data. The app captures HR data before and after strategy implementation, allowing for calculation of physiological stress reduction based on each strategy. These data allow for the calculation of the “Top 3 Strategies” for each child. These are included in push notifications linked with HR alarms, accessible via an app dashboard, and sent to teams on a daily or weekly basis via email, so that everyone can stay up to date with which strategies work best for each child. Thus, the technology gives teachers a tool to assess strategy effectiveness in restoring emotion regulation. This is adaptive and will allow teachers to tailor future use of strategies, thus supporting personalized treatment.

**Figure 3:**
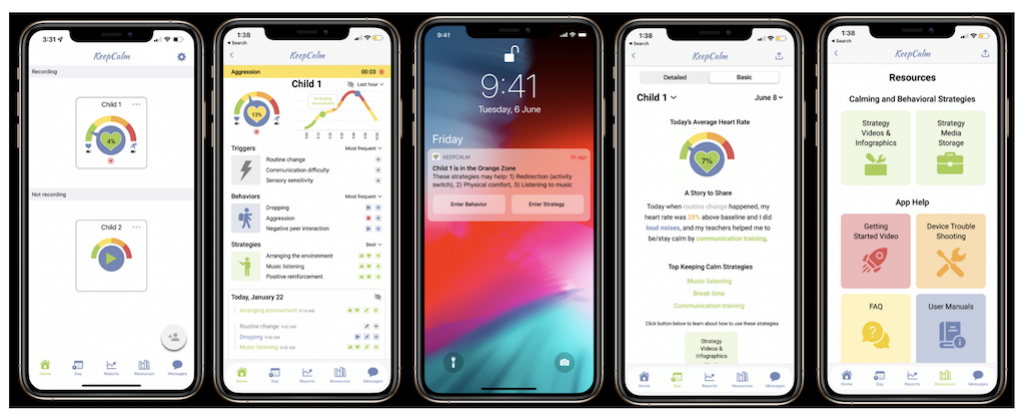
KeepCalm app design.

This app is aligned with the strategic plan of the Interagency Autism Coordinating Committee [62], which calls for maximizing the potential for technology-based intervention supports to improve the lives of individuals on the autism spectrum and developing technology-based treatments deployable in community settings to increase treatment access. In addition, our previous pilot data from parents and children show that they think that an app using HR signaling could be “extremely useful” for managing stress in students on the autism spectrum; they “strongly agree” that such a system would help prevent challenging behaviors, and they “strongly agree” that this offers advantages over current practices [63]. Figure 3 shows the main features and design of the KeepCalm app.

The KeepCalm app offers several features designed to make it easy to know when and how to intervene with children to support their proactive coping and emotion regulation development.

#### HR Stress Zone Tracking

The KeepCalm app pairs with selected commercially available HR trackers (refer to the *HR Trackers* section) that children wear to allow moment-by-moment physiological arousal tracking by educators. After recording a baseline HR for each child, educators are given pop-up notifications to alert them about moderate stress levels, along with individualized top strategies that can be used to reduce their stress. As HR is an index of stress levels [22-24], an increase in HR may index rising stress in children. HR above baseline (ie, HR at rest) is arranged in a stress zone rainbow in the KeepCalm app, with green representing baseline, yellow representing small increase in HR above baseline (+10%), orange representing medium increase in HR above baseline (+20%), and red representing large increase above baseline (+30%). Educators can track the child’s HR, so that they can intervene at moderate stress levels (ie, orange-zone HR) to prevent challenging behaviors associated with high stress levels or HR of approximately 30% above baseline [30] (ie, red-zone HR). In doing so, the educator creates emotion regulation learning opportunities for the child. Increase in HR may occur for other reasons, including increased physical exertion; however, accelerometry data movement thresholds ensure that increases in HR owing to child movement are not reflected in the HR stress zone tracking (refer to the *Offline Mode* section).

#### Individualized Child Behavior, Skills, Triggers, and Strategies Tracking

The KeepCalm app is designed to help educators and parents track children’s individualized education programs in a centralized manner. Teams can create custom profiles for each child for individualized tracking of challenging behaviors, skills, behavioral triggers and antecedents, and strategies for that child and sync data entry across the team.

#### Top Strategy Notifications

The KeepCalm app offers individualized “top strategy” suggestions from an up-to-date list of evidence-based strategies for supporting emotion regulation and preventing challenging behaviors in children on the autism spectrum. The top strategies are individualized for the child based on previous data collection and HR tracking and sent out when a child enters the orange or red stress zones.

#### Offline Mode

“Offline mode” is activated when a child’s average movement (acceleration) over the past 5 seconds is ≥1 m/s^2^. This threshold was selected to screen out the effect of vigorous movement on the top strategy notifications triggered by the child entering the orange or red stress zones.

#### Evidence-Based Strategy Resources

The app provides resources (infographics and videos) about the implementation of evidence-based strategies to help support emotion regulation and prevent challenging behaviors in children on the autism spectrum. These strategies are based on the research team’s comprehensive systematic review of the literature [64].

#### Day View

The KeepCalm app includes a “Day View” tab with a daily report including 2 versions. The first is a detailed day view that includes each data entry in list and figure format, and the second is a summary day view that includes a narrative description of the day including the top strategies used that day. This downloadable report is autogenerated at the end of each day and sent to the team via pop-up notification to support frequent team communication.

#### Custom Reports

The KeepCalm app includes a “Reports” tab where teams can produce clear and concise tables and figures to present data about each child’s challenging behaviors, skills, behavioral triggers and antecedents and strategies for upcoming education team meetings and to share with parents. These downloadable reports may be used to help with noticing trends and for evidence-based programming to support children’s development.

#### Functional Behavioral Assessment Mode

The KeepCalm app includes a functional behavioral assessment mode to help teams collect data to determine the function of or reason for new or emerging challenging behaviors, to help decide on strategies for these behaviors. Behavioral analysts can be added to the child’s KeepCalm team for easy sharing of functional behavioral assessment data.

#### Team Messaging

The KeepCalm app offers a team messaging platform to aid team communication, which is important for consistency of strategy use across school teams and at home. Teams can also contact the app development team in real time for troubleshooting.

To sign up to the app, school personnel and parents will enter their name, email, phone numbers, and their child’s nickname or ID, so that they can identify the child. Teams will record triggers, behavior and skills, and strategies in the app, and HR and accelerometry data will be recorded from child participants. Only the child’s team members and the research team will have access to this information. The app does not collect any other information from participants’ mobile devices.

#### HR Trackers

We will use 2 HR tracker models previously tested for accuracy, reliability, validity, and comfort [55]—the Mio Fuse (Mio Global) wristband and Polar H7 (POLAR) chest strap. These models are highly accurate (correlation with wired electrocardiogram: Mio Fuse: mean *r*=0.91, 95% CI 0.882-0.929; Polar H7: mean *r*=0.99, 95% CI 0.987-0.991) [53,56] and reliable (they meet quality thresholds on spike rate—Mio Fuse: 100%; Polar H7: 87.4%-89.4% and sampling fidelity—Mio Fuse: 96.2%-97.1%; Polar H7: 96.6%-100%) [55] for measuring HR and HR variability. We found that children on the autism spectrum rated both devices in the comfortable range [55], and in our pilot study, they were able to wear one of the 2 HR trackers during 5 school days.

#### Accelerometer

We will also use belt-clip accelerometers (mBient MetaMotionRL or another suitable model) to measure child movement, so that stress zone alerts are not sent out during period of high child movement but instead put the child’s notifications in “offline mode” (refer to the *Offline Mode* section for more details). In our pilot study, teachers were asked to use the mBient trackers with child participants during 5 school days.

### Measures

#### Primary Outcome Measures

##### Usability

Teacher and parent perceptions of usability will be measured using the System Usability Scale [65]. The scale consists of 10 items rated on a 5-point Likert scale ranging from 1 (“Strongly disagree”) to 5 (“Strongly agree”). Responses are transformed to obtain a total value from 0 to 100, with higher scores indicating greater perceptions of usability. This scale will be administered at the end of the trial, 3 months after baseline.

##### Feasibility

Feasibility of the app will be assessed through teacher and parent report using the Feasibility of Intervention Measure [66]. The measure consists of 4 items rated on a 5-point Likert scale ranging from 1 (“Completely disagree”) to 5 (“Completely agree”). The score is calculated by summing all items to yield a total score ranging from 4 to 20, with higher scores indicating greater feasibility of the app. Participants will complete this scale at the end of the trial, 3 months after baseline.

##### Acceptability

Acceptability of the app will be measured through teacher and parent report using the Acceptability of Intervention Measure [66]. The measure consists of 4 items rated on a 5-point Likert scale ranging from 1 (“Completely disagree”) to 5 (“Completely agree”). The score is calculated by summing all items to yield a total score ranging from 4 to 20, with higher scores indicating greater acceptability of the app. Participants will complete this scale at the end of the trial, 3 months after baseline.

##### Appropriateness

Appropriateness of the app will be assessed through teacher and parent report using the Intervention Appropriateness Measure [66]. The measure consists of 4 items rated on a 5-point Likert scale ranging from 1 (“Completely disagree”) to 5 (“Completely agree”). The score is calculated by summing all items to yield a total score ranging from 4 to 20, with higher scores indicating greater feasibility of the app. Participants will complete this scale at the end of the trial, 3 months after baseline.

##### Interview Feedback

Following participation in the study, teachers, aides, and parents will complete a 30-minute interview, where we will gather more context about the usability, feasibility, acceptability, and appropriateness of the KeepCalm app. We will also gather feedback, suggestions, and general impressions about the app. This feedback will also focus on the issue of alarm fatigue, that is, if teachers were overwhelmed, annoyed, or otherwise felt negatively toward the number of notifications received from the app during the day.

#### Secondary Outcome Measures

##### Proximal Preliminary Efficacy Outcomes

*Clinical decision support success*: We will additionally obtain information from teachers to measure the clinical decision support success of the app. This will be calculated from the proportion of alarms and emotion regulation strategy push notifications that result in timely implementation, as measured through in-class observation sessions (if possible, given the COVID-19–related school policies and teacher preferences) and remotely through data entry on the app.

*False positives and false negatives*: False positives and false negatives will be calculated as the proportion of alarms that appear to not be associated with an oncoming challenging behavior (eg, child movement) or challenging behaviors that occurred without an alarm, respectively. This will be measured through in-class observation sessions (if possible, given the COVID-19–related school policies and teacher preferences) and remotely through data entry on the app.

##### Distal Preliminary Efficacy Outcomes

*Challenging behaviors:* Challenging behaviors will be assessed using 3 measures: 2 measuring challenging behavior severity—the School Situations Questionnaire (SSQ [67]), completed by teachers, and the Open-Source Challenging Behavior Scale [68], completed by parents—and 1 measure of challenging behavior severity—the Institute for Basic Research Modified Overt Aggression Scale (IBR-MOAS [69]), completed by teachers and parents.

The SSQ assesses challenging behaviors within the school context. The scale assesses both the presence and the severity of challenging behaviors, with items assessing whether a given behavior occurs for a child and the severity ratings. The scale yields 2 summary scores. The first is the number of problems score, which is a sum of the number of challenging behaviors endorsed, ranging from 0 to 8, with higher scores indicating greater number of problems endorsed. The second is the mean severity, calculated as the mean severity for the items endorsed, ranging from 1 to 9, with higher numbers indicating more severe problems (1-3=mild severity, 4-6=moderate severity, and 7-9=significant severity). Participants will complete this measure at baseline and 3 months after baseline.

The Open-Source Challenging Behavior Scale measures the severity of challenging behavior episodes across contexts (eg, school and home), from the parent’s perspective. The scale includes 18 questions rated on a 5-point Likert scale (1=not at all a problem, 2=mild problem, 3=moderate problem, 4=severe problem, and 5=very severe problem). Participants will complete this measure at baseline and 3 months after baseline.

The IBR-MOAS measures the frequency and severity of aggressive behavior episodes. The scale is divided into five subscales: (1) verbal aggression toward others, (2) verbal aggression toward self, (3) physical aggression against other people, (4) physical aggression against objects, and (5) physical aggression against self. Items are rated on frequency on a 5-point Likert scale: never=never happens, rarely=averages about once a year to once a month, sometimes=averages about several times a month to several times per week, often=averages about daily to several times a day, and U=used to happen but not in the past year. For our analyses, we will code U ratings as equivalent to never. Unlike the original Overt Aggression Scale and other modified versions, a weighted scoring system was not used. We will adapt the measure to include other challenging behaviors of interest (eg, escape, climbing furniture, and meltdowns). Participants will complete this measure at baseline and 3 months after baseline.

*Emotion dysregulation:* Emotion dysregulation will be measured through parent and teacher reports using the Emotion Dysregulation Inventory (EDI), either the original version [70-72] or the young child version [73], depending on the child’s age. The scales consist of 30 items (original version) or 22 items (young child version) for raters to indicate how much of a problem certain emotions and behaviors are for the individual, being rated on a 5-point Likert scale ranging from 0 (“Not at all”) to 4 (“Very severe”). The EDI produces scores for reactivity (rapidly escalating, intense, and poorly regulated negative affect) and dysphoria (sadness, low positive affect, and unease). Raw scores are summed and converted to standardized scores with clinically elevated cutoffs, based on a large autism sample, and scoring information from a US census–matched sample of 1000 youths provides clinical cutoffs. Participants will complete this measure at baseline and 3 months after baseline.

#### Technical Outcomes

*Artifact removal:* The total number of “artifacts”—interbeat HR intervals (also known as RR intervals) >200 ms [55] detected during the testing period will be calculated throughout study participation.

*Offline mode activations*: The proportion of time for which “offline mode” (refer to the Offline Mode section for more details) is activated over the testing week based on the accelerometry data threshold will be calculated throughout study participation.

#### Adverse Events

We will use a brief Adverse Events Checklist to be administered to teachers and parents in the treatment group weekly throughout the trial, which may include items related to teachers experiencing stress triggered by monitoring their student’s stress levels. This checklist will be reviewed by the expert advisory board for the study before trial commencement.

### Ethics Approval

The trial is approved by the University of Pennsylvania institutional review board (829690) and the School District of Philadelphia research review committee (2022-12-1071).

### Procedure

The trial is registered at ClinicalTrials.gov (NCT05277194). After screening for eligibility, the 20 teams (at a minimum, teachers-parents and child dyads; at a maximum, teachers-parents and child-aide [or other educational team member] triads) will be randomized to the waitlist (n=10, 50%) or treatment group (n=10, 50%). The treatment (and later, the waitlist) group will use the app for 3 months. After a dyad or triad has signed up for the study, participants will complete a conferencing software-based intake meeting, during which they will be individually briefed about the KeepCalm app and complete the baseline assessments.

School personnel will then complete an in-app training session either remotely or in the classroom, during which they will receive access to the KeepCalm app, HR tracker, and accelerometer. The research team will also show or instruct teachers about how to put the HR tracker and accelerometer on the child or assist or instruct the child in putting them on themselves. Participants will download the app onto their own phones or, if they do not have an iPhone, an iPhone will be lent to them for their participation in the project. A member of the research team will also help the school personnel to customize the app for their student by showing them how to add the child’s profile and their triggers, behaviors and skills, and strategies. Standard but editable lists will be programmed into the app for each student, including evidence-based proactive and reactive strategies for reducing challenging behaviors and supporting emotion regulation [64]. All teachers recruited will have had professional development and in-class coaching in these strategies by coaches in our center. The research team will administer a fidelity assessment tool to teachers to understand their proficiency in implementing these strategies and provide additional coaching or resources for strategies, as needed.

Following this training, school personnel participants will begin testing the app within the classroom. They will initially record at least one baseline HR of the child, which will be used to calibrate the app’s stress zone notifications for the child. Baseline HR is recorded over a 5-minute period, during which the child will be engaged in a relaxing activity. We recommend that teachers take 2 to 3 baseline measurements to ensure accuracy. Students will then wear the HR tracker and accelerometer each day during the 3-month testing period. School personnel will be able to track the student’s HR and movement; enter child data about triggers and antecedents, challenging behaviors and skills, and intervention strategies; view the daily report; export the child’s data; and message others on the student’s team. School personnel will receive SMS text message support from the study team during the testing period and will be able to ask questions and troubleshoot any issues that arise during this period. Parents will be able to view the autogenerated daily report for the child, export and build figures of the child’s data, access the intervention strategy resources, and use the app’s messaging features. The research team will remotely monitor each participant’s use of the app during their respective testing period.

Following the app testing period, teachers, aides, and parents will provide feedback about their experience with the app during a brief interview with a member of the research team that will cover various themes including usability, acceptability, feasibility, appropriateness, and any adverse event that may have occurred (the latter will be in addition to the previously mentioned Adverse Events Checklist that will be administered weekly throughout the trial). Measures will be taken at eligibility determination, baseline, throughout the trial, and after the trial as per Table 1. A demographic survey will also be administered at baseline, including a question on the child’s current medications, as some are known to affect HR. If possible, given the COVID-19–related school policies and teacher preferences, the research team will perform in-class observations to obtain a first-hand look at the use of the KeepCalm app in the classroom and to track clinical decision support success and false positives and false negatives (as described in the *Measures* section).

**Table 1:** Timeline for data collection.

Construct and measure			Respondent or assessor		Timeline												
					Baseline					Throughout the trial (3 months)					After the trial		
					WL^a^		TX^b^		WL			TX		WL			TX
**Eligibility**																	
	SCQ^c^	Parent		✓		✓											
**IQ**																	
	Any standardized IQ battery, as reported on the child’s IEP^d^ (if not available, Stanford Binet ABIQ^e^)	Investigator, if required		✓		✓											
**Primary outcome measures**																	
	SUS^f^ (usability)	Teacher and parent														✓	
	AIM^g^ (acceptability)	Teacher and parent														✓	
	FIM^h^ (feasibility)	Teacher and parent														✓	
	IAM^i^ (appropriateness)	Teacher and parent														✓	
	Qualitative interview	Teacher, aide, and parent														✓	
**Technical reliability**																	
	Artifact removal success	Investigators (app data metrics)									✓						
	Offline mode activations	Investigators (app data metrics)									✓						
**Proximal preliminary efficacy**																	
	Implementation success	Investigators (classroom observation)									✓						
	False positives and false negatives	Investigators (classroom observation)									✓						
**Distal preliminary efficacy**																	
	SSQ^j^ (CB^k^ severity)	Teachers		✓		✓							✓			✓	
	OS-CBS^l^ (CB severity)	Parents		✓		✓							✓			✓	
	IBR-MOAS^m^ (CB frequency)	Teachers and parents		✓		✓							✓			✓	
	EDI^n^ (dysregulation)	Teachers and parents		✓		✓							✓			✓	
**Adverse events**																	
	Adverse events checklist	Teachers and parents									✓					✓	

^a^WL: waitlist group.

^b^TX: treatment group.

^c^SCQ: Social Communication Questionnaire.

^d^IEP: Individual Education Program.

^e^ABIQ: Abbreviated Battery IQ.

^f^SUS: System Usability Scale.

^g^AIM: Acceptability of Intervention Measure.

^h^FIM: Feasibility of Intervention Measure.

^i^IAM: Intervention Appropriateness Measure.

^j^SSQ: School Situations Questionnaire.

^k^CB: challenging behavior.

^l^OS-CBS: Open-Source Challenging Behavior Scale.

^m^IBR-MOAS: Institute for Basic Research Modified Overt Aggression Scale.

^n^EDI: Emotion Dysregulation Inventory.

### Data Analysis

#### Primary Outcomes

This trial is designed to test usability, acceptability, appropriateness, and feasibility data rather than efficacy; therefore, primary outcome data analyses will use descriptive statistics of the survey measures on usability (System Usability Scale), acceptability (Acceptability of Intervention Measure), feasibility (Feasibility of Intervention Measure), and appropriateness (Intervention Appropriateness Measure) of the KeepCalm app, as outlined in the *Measures* section.

We will also examine usability, acceptability, feasibility, and appropriateness through a qualitative interview and examine barriers to and facilitators of using the KeepCalm app in classrooms with students on the autism spectrum. Audio recordings of the interviews will be transcribed, and all transcripts will be loaded into Dedoose for data management and analysis. Thematic analysis will be guided by an integrated approach that includes identification of a priori attributes of interest (usability, acceptability, feasibility, and appropriateness). This integrated approach uses an inductive process of iterative coding to identify recurrent themes, categories, and relationships in qualitative data. After exploring the data, a comprehensive coding scheme will be developed and applied to produce a descriptive analysis. A team of coders will separately code the transcripts and compare the results to assess the reliability of the coding scheme. Any disagreements in coding will be resolved by team consensus. After the coding scheme is finalized, coders will be required to code 2 transcripts in a row at a high level of agreement with the lead coder (*k*>0.70) before independent coding of the rest of the transcripts. Following this, the lead coder will code 20% of the transcripts in duplicate for the reporting of interrater reliability.

Quantitative and qualitative data about usability, acceptability, feasibility, and appropriateness will be integrated by examining the qualitative data from the participants who responded in the lowest versus highest quartile on the quantitative measures of each construct. Data analysis will inform our understanding of the usability, acceptability, feasibility, and appropriateness of the KeepCalm app and will provide a list of feature updates to be included in the app before the following fully powered randomized controlled trial.

#### Secondary Outcomes

To examine technical reliability, we will use descriptive statistics of artifact removal success and offline mode activations. To examine proximal preliminary efficacy, we will use descriptive statistics of clinical decision support success rates and false positive and negative rates. To examine distal preliminary efficacy, we will also conduct a preliminary examination of the efficacy of KeepCalm on challenging behavior (SSQ and IBR-MOAS) and emotion dysregulation (EDI) using an exploratory (underpowered) repeated measures ANOVA to assess the 2 groups (waitlist and treatment) at baseline and posttrial periods. Given the sample size, this analysis will be underpowered.

#### Power

For the study’s pilot randomized controlled trial, the analyses will focus on feasibility, usability, and implementation (descriptive) data but will include some preliminary efficacy inferential analyses. In this study, 50% (10/20) of the teams will be randomized to the treatment condition and 50% (10/20) of the teams will be randomized to the waitlist control condition. The sample size was not based on power analysis but rather a need to obtain pilot data that will support an app for a fully powered trial.

## Results

We anticipate that recruitment for the randomized controlled trial will begin in September 2023. The analysis of data will begin following the trial, which we expect to be completed in the second half of 2024. We expect to publish the results of the trial in 2025.

## Discussion

In this paper, we described the protocol of an innovative pilot randomized waitlist-controlled field trial to investigate the usability, acceptability, feasibility, and preliminary efficacy of an app to detect and manage challenging behaviors in children on the autism spectrum. We aim to address current barriers that limit the use of emotion regulation strategies with children on the autism spectrum by developing this evidence-based strategy implementation tool based on objective, physiological measurements. This study offers several innovations to be tested. First, it tests an app that integrates physiological stress monitoring, to track children’s internal stress that is less observable, with real-time intervention suggestions. Second, the intervention suggestions provided by the app are individualized to each child. It reminds teachers about child-specific emotion regulation strategies and provides in-app resources about evidence-based strategies to help parents and teachers learn strategies that may be more effective to use with the child. Finally, the app will analyze which strategies work best for each individual child based on physiological stress reduction data, teacher’s ratings of strategy effectiveness, and child’s behavioral data.

The mHealth KeepCalm app is an app designed to target challenging behaviors in children on the autism spectrum using biosensing technology. The main limitation we anticipate facing in this trial is that, owing to the small sample size of only 20 teams, we will lack sufficient power to determine the efficacy of the app in preventing and reducing challenging behaviors. However, this trial will be aimed at gathering usability, acceptability, feasibility, and appropriateness data and information about barriers and facilitators to prepare for a large randomized controlled trial to gather data about effectiveness, which will be conducted later.

We are not aware of any other digital health app for children on the autism spectrum that uses physiological biosensing to monitor physiological arousal and support the implementation of evidence-based practices for children on the autism spectrum. Although there are many other types of technological innovations to support individuals on the autism spectrum and despite the range of digital mental health apps that are commercially available, there is little evidence about their efficacy [74]. However, as mentioned previously, >95% of them have not actually been studied, and there is a lack of studies in this field regarding efficacy, especially relative to studies of web-based mental health programs [44,45].

Current implementation of strategies to manage challenging behaviors or emotion dysregulation by school teams is limited by difficulties in detecting rising stress levels in children on the autism spectrum who struggle to communicate their emotions, chaotic and stressful classroom environments that make it difficult to recall the best emotion regulation strategies per student, and difficulty in tracking the effectiveness of these strategies for ongoing program refinement. The mHealth KeepCalm app seeks to address these barriers with new biosensing technology and takes a personalized medicine approach to reduce the impact of significant challenging behaviors in children on the autism spectrum.

## Appendix

Multimedia Appendix 1 Peer-review report from the Center for Scientific Review Special Emphasis Panel - National Institute of Mental Health (National Institutes of Health, USA).

## Abbreviations

AUC: area under the curve
EDI: Emotion Dysregulation Inventory
HR: heart rate
IBR-MOAS: Institute for Basic Research Modified Overt Aggression Scale
JITAI: just-in-time adaptive intervention
mHealth: mobile health
SSQ: School Situations Questionnaire

## Data Availability

Data sharing is not applicable to this paper, as no trial data sets have been generated or analyzed at this time. Data that will be collected in the trial described in this paper, which will be published in a future manuscript, will adhere to the established rules set forth by the National Institutes of Health for public sharing of data.

## References

[1] Machalicek W, O’Reilly MF, Beretvas N, Sigafoos J, Lancioni GE (2007). A review of interventions to reduce challenging behavior in school settings for students with autism spectrum disorders. Res Autism Spectrum Disord.

[2] Rojahn J, Matson J, Lott D, Esbensen A, Smalls Y (2001). The Behavior Problems Inventory: an instrument for the assessment of self-injury, stereotyped behavior, and aggression/destruction in individuals with developmental disabilities. J Autism Dev Disord.

[3] Matson JL, Rivet TT (2008). Characteristics of challenging behaviours in adults with autistic disorder, PDD-NOS, and intellectual disability. J Intellect Dev Disabil.

[4] Hattier MA, Matson JL, Belva BC, Horovitz M (2011). The occurrence of challenging behaviours in children with autism spectrum disorders and atypical development. Dev Neurorehabil.

[5] Lecavalier L, Leone S, Wiltz J (2006). The impact of behaviour problems on caregiver stress in young people with autism spectrum disorders. J Intellect Disabil Res.

[6] Hastings RP, Brown T (2002). Coping strategies and the impact of challenging behaviors on special educators' burnout. Mental Retardation.

[7] Siegel M, Gabriels RL (2014). Psychiatric hospital treatment of children with autism and serious behavioral disturbance. Child Adolesc Psychiatr Clin N Am.

[8] Cohen I, Yoo J, Goodwin M, Moskowitz L (2011). Assessing challenging behaviors in autism spectrum disorders: prevalence, rating scales, and autonomic indicators. International Handbook of Autism and Pervasive Developmental Disorders.

[9] Mazefsky CA, Herrington J, Siegel M, Scarpa A, Maddox BB, Scahill L, White SW (2013). The role of emotion regulation in autism spectrum disorder. J Am Acad Child Adolesc Psychiatry.

[10] Thompson RA (2008). Emotion regulation: a theme in search of definition. Monographs Society Res Child Develop.

[11] Siegel M (2018). Aggression and self-injury: research needs for the severely affected end of the spectrum. Proceedings of the IACC meeting.

[12] Siegel M (2018). Arousal, emotion regulation and challenging behaviors: insights from the autism inpatient collection. Simons Foundation.

[13] Maddox BB, Cleary P, Kuschner ES, Miller JS, Armour AC, Guy L, Kenworthy L, Schultz RT, Yerys BE (2018). Lagging skills contribute to challenging behaviors in children with autism spectrum disorder without intellectual disability. Autism.

[14] Nuske HJ, McGhee Hassrick E, Bronstein B, Hauptman L, Aponte C, Levato L, Stahmer A, Mandell DS, Mundy P, Kasari C, Smith T (2019). Broken bridges-new school transitions for students with autism spectrum disorder: a systematic review on difficulties and strategies for success. Autism.

[15] Doehring P, Reichow B, Palka T, Phillips C, Hagopian L (2014). Behavioral approaches to managing severe problem behaviors in children with autism spectrum and related developmental disorders: a descriptive analysis. Child Adolesc Psychiatr Clin N Am.

[16] Otten K, Tuttle J (2010). How to Reach and Teach Children with Challenging Behavior (K-8) Practical, Ready-to-Use Interventions That Work.

[17] Sugai G, Horner RH, Dunlap G, Hieneman M, Lewis TJ, Nelson CM, Scott T, Liaupsin C, Sailor W, Turnbull AP, Turnbull HR, Wickham D, Wilcox B, Ruef M (2016). Applying positive behavior support and functional behavioral assessment in schools. J Positive Behav Intervent.

[18] Nuske HJ, Vivanti G, Dissanayake C (2013). Are emotion impairments unique to, universal, or specific in autism spectrum disorder? A comprehensive review. Cogn Emot.

[19] Bradley MM, Miccoli L, Escrig MA, Lang PJ (2008). The pupil as a measure of emotional arousal and autonomic activation. Psychophysiology.

[20] Ioannou S, Gallese V, Merla A (2014). Thermal infrared imaging in psychophysiology: potentialities and limits. Psychophysiology.

[21] Stern R, Ray W, Quigley K (2000). Psychophysiological Recording.

[22] Thayer JF, Ahs F, Fredrikson M, Sollers JJ, Wager TD (2012). A meta-analysis of heart rate variability and neuroimaging studies: implications for heart rate variability as a marker of stress and health. Neurosci Biobehav Rev.

[23] Schiweck C, Piette D, Berckmans D, Claes S, Vrieze E (2018). Heart rate and high frequency heart rate variability during stress as biomarker for clinical depression. A systematic review. Psychol Med.

[24] Bali A, Jaggi A (2015). Clinical experimental stress studies: methods and assessment. Rev Neurosci.

[25] Groden J, Cautela J, Prince S, Berryman J (1994). The impact of stress and anxiety on individuals with autism and developmental disabilities. Behavioral Issues in Autism. Current Issues in Autism.

[26] Romanczyk R, Gillis J (2006). Autism and the physiology of stress and anxiety. Stress and Coping in Autism.

[27] Romanczyk R (1986). Self-injurious behavior: conceptualization, assessment, and treatment. Adv Learn Behav Disabil.

[28] Barrera FJ, Violo RA, Graver EE (2007). On the form and function of severe self-injurious behavior. Behav Intervent.

[29] Freeman R, Grzymala-Busse J, Riffel L, Schroeder S (2001). Analyzing the relation between heart rate, problem behavior, and environmental events using data mining system LERS. Proceedings of the 14th IEEE Symposium on Computer-Based Medical Systems.

[30] Nuske HJ, Finkel E, Hedley D, Parma V, Tomczuk L, Pellecchia M, Herrington J, Marcus SC, Mandell DS, Dissanayake C (2019). Heart rate increase predicts challenging behavior episodes in preschoolers with autism. Stress.

[31] Goodwin MS, Mazefsky CA, Ioannidis S, Erdogmus D, Siegel M (2019). Predicting aggression to others in youth with autism using a wearable biosensor. Autism Res.

[32] Jahromi L, Meek S, Ober-Reynolds S (2012). Emotion regulation in the context of frustration in children with high functioning autism and their typical peers. J Child Psychol Psychiatry.

[33] Rieffe C, Oosterveld P, Terwogt MM, Mootz S, van Leeuwen E, Stockmann L (2011). Emotion regulation and internalizing symptoms in children with autism spectrum disorders. Autism.

[34] Samson AC, Hardan AY, Lee IA, Phillips JM, Gross JJ (2015). Maladaptive behavior in autism spectrum disorder: the role of emotion experience and emotion regulation. J Autism Dev Disord.

[35] Samson AC, Huber O, Gross JJ (2012). Emotion regulation in Asperger's syndrome and high-functioning autism. Emotion.

[36] Samson AC, Wells WM, Phillips JM, Hardan AY, Gross JJ (2015). Emotion regulation in autism spectrum disorder: evidence from parent interviews and children's daily diaries. J Child Psychol Psychiatry.

[37] Mazefsky CA, Borue X, Day TN, Minshew NJ (2014). Emotion regulation patterns in adolescents with high-functioning autism spectrum disorder: comparison to typically developing adolescents and association with psychiatric symptoms. Autism Res.

[38] Domitrovich CE, Pas ET, Bradshaw CP, Becker KD, Keperling JP, Embry DD, Ialongo N (2015). Individual and school organizational factors that influence implementation of the PAX good behavior game intervention. Prev Sci.

[39] Wehby JH, Maggin DM, Moore Partin TC, Robertson R (2011). The impact of working alliance, social validity, and teacher burnout on implementation fidelity of the good behavior game. School Mental Health.

[40] Nahum-Shani I, Smith S, Spring B, Collins L, Witkiewitz K, Tewari A, Murphy S (2018). Just-in-time adaptive interventions (JITAIs) in mobile health: key components and design principles for ongoing health behavior support. Ann Behav Med.

[41] Gustafson DH, McTavish FM, Chih M, Atwood AK, Johnson RA, Boyle MG, Levy MS, Driscoll H, Chisholm SM, Dillenburg L, Isham A, Shah D (2014). A smartphone application to support recovery from alcoholism: a randomized clinical trial. JAMA Psychiatry.

[42] Juarascio A, Srivastava P, Presseller E, Clark K, Manasse S, Forman E (2021). A clinician-controlled just-in-time adaptive intervention system (CBT+) designed to promote acquisition and utilization of cognitive behavioral therapy skills in bulimia nervosa: development and preliminary evaluation study. JMIR Form Res.

[43] Clausen CE, Leventhal BL, Nytrø Ø, Koposov R, Westbye OS, Røst TB, Bakken V, Koochakpour K, Thorvik K, Skokauskas N (2020). Testing an individualized digital decision assist system for the diagnosis and management of mental and behavior disorders in children and adolescents. BMC Med Inform Decis Mak.

[44] Lecomte T, Potvin S, Corbière M, Guay S, Samson C, Cloutier B, Francoeur A, Pennou A, Khazaal Y (2020). Mobile apps for mental health issues: meta-review of meta-analyses. JMIR Mhealth Uhealth.

[45] Torous J, Nicholas J, Larsen ME, Firth J, Christensen H (2018). Clinical review of user engagement with mental health smartphone apps: evidence, theory and improvements. Evid Based Ment Health.

[46] Gould JD, Lewis C (1985). Designing for usability: key principles and what designers think. Commun ACM.

[47] (1986). User Centered System Design New Perspectives on Human-computer Interaction.

[48] (2017). Participatory Design & Health Information Technology.

[49] Schnall R, Rojas M, Bakken S, Brown W, Carballo-Dieguez A, Carry M, Gelaude D, Mosley JP, Travers J (2016). A user-centered model for designing consumer mobile health (mHealth) applications (apps). J Biomed Inform.

[50] Yen P, Bakken S (2012). Review of health information technology usability study methodologies. J Am Med Inform Assoc.

[51] Rutter M, Bailey A, Lord C (2003). The Social Communication Questionnaire.

[52] Roid G (2003). Stanford-Binet Intelligence Scales, Fifth Edition (SB:V).

[53] Wang R, Blackburn G, Desai M, Phelan D, Gillinov L, Houghtaling P, Gillinov M (2017). Accuracy of wrist-worn heart rate monitors. JAMA Cardiol.

[54] Goodwin MS (2008). Enhancing and accelerating the pace of autism research and treatment. Focus Autism Other Dev Disabl.

[55] Herrington J, Nuske H, Kushleyeva Y, Bonafide C, Masino A, Pennington J (2023). Common Sense Validation of Wearable Heart Rate Devices for Psychophysiological Research. under review.

[56] Wijaya A, Prihatmanto A, Wijaya R (2016). Shesop healthcare: android application to monitor heart rate variance, display influenza and stress condition using Polar H7. arXiv.

[57] Nuske HJ, Kushleyeva Y, Forsyth D, Pennington JW, Masino AJ, Finkel E, Bhattacharya A, Mandell DS, Bonafide CP, Herrington JD (2019). Comfortable, valid and reliable? Measuring stress in children with asd using select consumer-grade heart rate trackers. Proceedings of the INSAR 2019.

[58] Saleem JJ, Patterson ES, Militello L, Anders S, Falciglia M, Wissman JA, Roth EM, Asch SM (2007). Impact of clinical reminder redesign on learnability, efficiency, usability, and workload for ambulatory clinic nurses. J Am Med Inform Assoc.

[59] Saleem JJ, Patterson ES, Militello L, Render ML, Orshansky G, Asch SM (2005). Exploring barriers and facilitators to the use of computerized clinical reminders. J Am Med Inform Assoc.

[60] Hunt DL, Haynes RB, Hanna SE, Smith K (1998). Effects of computer-based clinical decision support systems on physician performance and patient outcomes: a systematic review. JAMA.

[61] Vashitz G, Meyer J, Gilutz H (2007). General practitioners' adherence with clinical reminders for secondary prevention of dyslipidemia. AMIA Annu Symp Proc.

[62] Interagency Autism Coordinating Committee (IACC) (2020). Interagency autism coordinating committee strategic plan for autism spectrum disorder 2018-2019 update. U.S. Department of Health and Human Services Interagency Autism Coordinating Committee.

[63] Nuske H, Pennington J, Goodwin M, Sultanik E, Mandell D (2020). A m-health platform for teachers of children with autism to support emotion regulation. Proceedings of the INSAR 2020.

[64] Nuske H, Ba BA, Khan K, Palermo E, Ajanaku B, Pellecchia M, Vivanti G, Mazefsky C, Brookman-Frazee L, McPartland J, Goodwin M, Mandell D (2023). Systematic review: emotion dysregulation and challenging behavior interventions for children andadolescents with autism with graded key evidence-based strategy recommendations. Res Sq.

[65] Brooke J (1996). SUS: a 'quick and dirty' usability scale. Usability Evaluation In Industry.

[66] Weiner BJ, Lewis CC, Stanick C, Powell BJ, Dorsey CN, Clary AS, Boynton MH, Halko H (2017). Psychometric assessment of three newly developed implementation outcome measures. Implement Sci.

[67] Altepeter TS, Breen MJ (2016). The home situations questionnaire (HSQ) and the school situations questionnaire (SSQ): normative data and an evaluation of psychometric properties. J Psychoeducational Assess.

[68] Frazier TW, Khaliq I, Scullin K, Uljarevic M, Shih A, Karpur A (2022). Development and psychometric evaluation of the open-source challenging behavior scale (OS-CBS). J Autism Dev Disord.

[69] Cohen IL, Tsiouris JA, Flory MJ, Kim S, Freedland R, Heaney G, Pettinger J, Brown WT (2010). A large scale study of the psychometric characteristics of the IBR Modified Overt Aggression Scale: findings and evidence for increased self-destructive behaviors in adult females with autism spectrum disorder. J Autism Dev Disord.

[70] Mazefsky CA, Day TN, Siegel M, White SW, Yu L, Pilkonis PA, AutismDevelopmental Disabilities Inpatient Research Collaborative (ADDIRC) (2018). Development of the emotion dysregulation inventory: a PROMIS®ing method for creating sensitive and unbiased questionnaires for autism spectrum disorder. J Autism Dev Disord.

[71] Mazefsky CA, Yu L, White SW, Siegel M, Pilkonis PA (2018). The emotion dysregulation inventory: psychometric properties and item response theory calibration in an autism spectrum disorder sample. Autism Res.

[72] Mazefsky CA, Yu L, Pilkonis PA (2021). Psychometric properties of the emotion dysregulation inventory in a nationally representative sample of youth. J Clin Child Adolesc Psychol.

[73] Day T, Northrup J, Mazefsky C (2022). A PROMIS®ing new measure for quantifying emotion dysregulation in toddlers and preschoolers: development of the emotion dysregulation inventory-young child. J Autism Dev Disord.

[74] Nuske HJ, Mandell DS (2021). Digital health should augment (not replace) autism treatment providers. Autism.

