# Supplementary material for: A Digital Mental Health App Incorporating Wearable Biosensing for Teachers of Children on the Autism Spectrum to Support Emotion Regulation: Protocol for a Pilot Randomized Controlled Trial
Source: JMIR Res Protoc. 2023 Jun 26;12:e45852. doi: 10.2196/45852 (PMC10337316; doi:10.2196/45852)
Supplement: Multimedia Appendix 1 [file resprot_v12i1e45852_app1.pdf]

SUMMARY STATEMENT
(Privileged Communication)

PROGRAM CONTACT:
Dr. Kathleen Anderson
301-443-5837
kanders1@mail.nih.gov

Release Date: 11/25/2019
Revised Date:

Application Number: 1 K01 MH120509-01A1

Principal Investigator

NUSKE, HEATHER J

Applicant Organization: UNIVERSITY OF PENNSYLVANIA

Review Group: ZRG1 BBBP-X (02)
Center for Scientific Review Special Emphasis Panel
Member Conflict: Child Psychopathology and Developmental Disabilities

Meeting Date: 11/21/2019
Council: JAN 2020
Requested Start: 04/01/2020
RFA/PA: PA19-127
PCC: BK-TK
Dual IC(s): HD

Project Title: Reducing Challenging Behaviors In Children With Autism Through Digital Health

SRG Action: Impact Score:14
Next Steps: Visit https://grants.nih.gov/grants/next_steps.htm
Human Subjects: 30-Human subjects involved - Certified, no SRG concerns
Animal Subjects: 10-No live vertebrate animals involved for competing appl.
Gender: 1A-Both genders, scientifically acceptable
Minority: 1A-Minorities and non-minorities, scientifically acceptable
Age: 2A-Only Children, scientifically acceptable

Project Year	Direct Costs Requested	Estimated Total Cost
1	168,440	180,659
2	172,642	185,166
3	176,321	189,112
4	180,111	193,177
TOTAL	697,514	748,115

ADMINISTRATIVE BUDGET NOTE: The budget shown is the requested budget and has not been adjusted to reflect any recommendations made by reviewers. If an award is planned, the costs will be calculated by Institute grants management staff based on the recommendations outlined below in the COMMITTEE BUDGET RECOMMENDATIONS section.

1K01MH120509-01A1 Nuske, Heather

RESUME AND SUMMARY OF DISCUSSION: This resubmission for a mentored research scientist development award is proposed by a developmental psychologist with interest in addressing emotion dysregulation and challenging behaviors in children with autism spectrum disorder (ASD) in community settings. The Candidate seeks additional career development experience in community-based interventions for challenging behaviors, qualitative methods, mHealth interventions, psycho-physiological measurement, and hybrid implementation/effectiveness designs. If successful, there will be increased understanding of the utility of the EMOtion Regulation application (EMORE app) as a means of reducing challenging behavior in the classroom setting. The resubmission was largely responsive to the previous review, although additional discussion might have been provided for issues regarding heart rate and how behavioral interventions will be chosen for a specific child. The Candidate is outstanding with multiple publications and funded grant awards. The primary mentor, Dr. Mandell, is an expert in community-based interventions and implementation research; other mentors provide complementary skills. The environments at the University of Pennsylvania (UPenn) and Children's Hospital of Philadelphia (CHOP) are outstanding. The career development plan is well-organized and has now been pared down sensibly in response to the previous review. The approach is well thought out with multiple rounds of product input, design, and re-design. The developmental phase of this resubmission, which seeks input from key stakeholders, is much improved. However, it is possible that heart rate may increase for other reasons (not specific to challenging behaviors) and thus an intervention may be implemented that is unneeded or not appropriate. Overall, this is an exceptional resubmission that has high potential to impact the field of ASD.

DESCRIPTION (provided by applicant): The project will develop and pilot test a personalized medicine mobile health EMOtion REgulation application (m-health EMORE app) that incorporates physiological stress measurement to support evidence-based practices for reducing challenging behavior in children with autism spectrum disorder (ASD). As much as 80% of children with ASD exhibit challenging behaviors that can have a devastating impact on personal and family well-being, contribute to teacher burnout and require frequent hospitalization. Evidence-based practices for reducing these behaviors emphasize uncovering triggers, yet parents and teachers often report that challenging behaviors surface without warning. Challenging behaviors caused by emotion dysregulation can be the most difficult to predict, as children with ASD often have difficulty communicating their distress before it results in challenging behavior. Exciting recent advances in digital technology now allow measurement of momentary emotion dysregulation, using physiological indices. Our pilot data from four separate samples demonstrate that increased heart rate predicts onset of challenging behavior in children with ASD. In order to tailor the m-health EMORE app to end users and avoid potential barriers to its adoption, in Aim 1, we will evaluate the acceptability, feasibility, and appropriateness of app, and the needs of educational teams in managing stress in children with ASD and challenging behaviors, by conducting interviews with teachers of children with ASD, parents of children with ASD and school administrators, and conducting structured in-class observations with teachers. Through the activities of Aim 2, we will improve our m-health EMORE app, building on our initial app prototype, in collaboration with our established research partner, the Translational Informatics Unit, Children's Hospital of Philadelphia, and our established community partner, the School District of Philadelphia. We will do this through 1) exploratory work on the specificity of heart rate increase to challenging behavior, on app clinical decision support timing, and on the association of app false positives and negatives to movement or child factors; 2) monthly advisory board meetings with expert stakeholders for app development guidance, and; 3) rapid-cycle prototyping of the app with 10 educational teams (i.e. 1-2 children with ASD, and their teacher and classroom aide, if they have one). This will allow for iterative improvement based on each user's experience. Through Aim 3, we will test the app for usability, acceptability, feasibility and appropriateness, as well as preliminary effectiveness with 30 educational teams in a randomized waitlist field trial over a 3-month period. Successful completion of these aims will result in a novel m-health app designed to help teachers support emotion regulation, and reduce or

prevent challenging behavior in children with ASD, using evidence-based strategies. These activities will lay the foundation for an R01 to evaluate the effectiveness of the m-health EMORE app in a full-scale randomized field trial. Our proposal aligns with the strategic plan of the Interagency Autism Coordinating Committee, to maximize the potential for technology-based interventions to improve the lives of people with ASD.

PUBLIC HEALTH RELEVANCE: School-based behavioral approaches to managing challenging behaviors in children with ASD are limited by three key factors: 1) children with ASD often have difficulties communicating their emotions; 2) it is challenging to implement evidence-based, personalized strategies for individual children, and; 3) it is difficult for teachers to track which strategies are successful for individual children. Our proposed personalized mobile-health emotion regulation application (m-health app) will pair heart rate tracking with digital tools to help reduce challenging behavior by supporting stress detection, reminding teachers of specific behavioral strategies and helping teachers to track progress.

CRITIQUE 1

Candidate: 1
Career Development Plan/Career Goals /Plan to Provide Mentoring: 1
Research Plan: 1
Mentor(s), Co-Mentor(s), Consultant(s), Collaborator(s): 1
Environment Commitment to the Candidate: 1

Overall Impact: This resubmitted K application comes from an exceptional Candidate with an exceptional mentoring team. Her training plan is ambitious but also well laid out and described, and by all accounts, this Candidate will be capable of achieving her training objectives. She seeks further training in community-based challenging behavior interventions, qualitative methods, development of m-Health applications, advanced psychophysiological analysis, and community-based effectiveness-implementation. The research plan is to directly test feasibility and utility of the EMOtion Regulation application (EMORE app) as a means of reducing challenging behavior in the classroom setting. The research plan is sound and clear and aligns well with the proposed training plan. Overall, the proposed project is likely to further the likelihood of long-term success in an already successful researcher who is early in her career.

1. Candidate

Strengths

- The Candidate is exceptionally well trained in areas relevant to her career goals. Additional training as part of this K is highly likely to position her as an independent researcher.

Weaknesses

- None noted by reviewer.

2. Career Development Plan/Career Goals & Objectives

Strengths

- The career development plan is clearly conceptualized and laid out in the application.
- There is an explicit plan for coordinating training activities with the different mentors.

Weaknesses

- None noted by reviewer.

3. Research Plan

Strengths

- The step wise approach to investigating the functionality of the EMORE app will provide information about its acceptability, feasibility, appropriateness, as well as preliminary data regarding effectiveness.
- Data generated from the project will serve as pilot data for a larger-scale test of the app.

Weaknesses

- None noted by reviewer.

4. Mentor(s), Co-Mentor(s), Consultant(s), Collaborator(s)

Strengths

- Like the Candidate, the mentoring team is also exceptional.

Weaknesses

- None noted by reviewer.

5. Environment and Institutional Commitment to the Candidate

Strengths

- Given the Candidate’s interest in implementation science in autism spectrum disorder (ASD), it is difficult to imagine a better environment or mentoring team.

Weaknesses

- None noted by reviewer.

Protections for Human Subjects:

Acceptable Risks and/or Adequate Protections

Data and Safety Monitoring Plan (Applicable for Clinical Trials Only):

- o Appropriate

Inclusion Plans:

- Sex/Gender: Distribution justified scientifically
- Race/Ethnicity: Distribution justified scientifically
- Inclusion/Exclusion Based on Age: Including ages <18; justified scientifically
- Appropriate

Vertebrate Animals:

Not Applicable (No Vertebrate Animals)

Biohazards:

Not Applicable (No Biohazards)

Training in the Responsible Conduct of Research

Comments on Format (Required):

Comments on Subject Matter (Required):

Comments on Faculty Participation (Required; not applicable for mid- and senior-career awards):

Comments on Duration (Required):

Comments on Frequency (Required):

Budget and Period of Support

Recommend as Requested

CRITIQUE 2

Candidate: 1
Career Development Plan/Career Goals /Plan to Provide Mentoring: 1
Research Plan: 2
Mentor(s), Co-Mentor(s), Consultant(s), Collaborator(s): 1
Environment Commitment to the Candidate: 1

Overall Impact: This career development award is proposed by a developmental psychologist with interest in addressing emotion dysregulation and challenging behaviors for children with autism spectrum disorder (ASD) in community settings. The Candidate has substantial experience in both the topic matter as well as in management of research projects. She seeks additional career development experience in community-based interventions for challenging behaviors, qualitative methods, mHealth interventions, psycho-physiological measurement, and hybrid implementation/effectiveness designs. The primary mentor, Dr. Mandell, is an expert in community-based interventions and implementation research; other mentors provide complementary skills. This was a very strong resubmission that responded well to reviewer critiques in the prior round, in nearly all areas. The career development plan was well-organized and has a strong mentor team. The plan was pared down sensibly in response to reviewer critiques. The Candidate herself is outstanding with multiple publications and funded grant awards. The research plan is of potentially high significance--the area of wearable biosensors generally is a fast-moving part of mental health research. The approach also is well thought out with multiple rounds of product input, design, and re-design. The developmental phase of this proposal, which seeks input from key stakeholders, is much improved. Although it is still somewhat of an open question whether heart rate variability (HRV) will be a key therapeutic indicator in ASD, this seems a worthy question to investigate, particularly in the context of a career development award where more could be learned about mHealth, research techniques, and wearable sensors more generally. The environment also is outstanding. As a result, this application was judged to be of high impact with only minor weaknesses.

1. Candidate

Strengths

- Candidate has personal and clinical experience in challenging behaviors in ASD.

- The Candidate has had a good record of scholarly productivity during her PhD and several postdocs, with multiple first-author publications and funded grants.
- She will have a faculty position at University of Pennsylvania that is not contingent on this K award; however, this K01 would allow her much more research independence.
- Referee letters from high-profile leaders in autism research; it is clear she has been a key contributor to research in Dr. Mandell's laboratory and is known to many in the field; there would be a good support network for her outside of her mentor team.

Weaknesses

- None noted by reviewer.

2. Career Development Plan/Career Goals & Objectives

Strengths

- This remains a very well-organized and articulate plan.
- The resubmission was very responsive to reviewer critiques to increase mentorship in mHealth and psychophysiology.
- The coursework and mentor meetings have been reduced which makes it seem more manageable.

Weaknesses

- It seems like some of the papers proposed do not clearly fit the research plan. However, translating some of the research done here into academic products may be challenging, since so much of it is product development, so it may be reasonable to seek out other avenues for papers.

3. Research Plan

Strengths

- The project is of high potential significance; interventions for emotional dysregulation and challenging behaviors in ASD are sorely needed.
- Aims and hypotheses are clearly stated.
- The plan to use physiologic data to predict challenging behaviors in ASD, in the classroom setting, is fairly innovative. There is potential for it to be useful for other conditions as well.
- They have already tested several wearable devices on children with ASD and found the heart rate (HR) and HRV to be acceptable. They also found that many children with ASD will wear the devices.
- There have been some design workshops with parents, people with ASD, and teachers to develop some initial parameters for the device. This resubmission improves the feasibility and acceptability of assessment proposed; it is now quite robust.
- The user-centered design approach to development of the app is a good approach in this situation, and the rapid-cycle prototyping will also lead to improvements to the device.
- The qualitative methods in Aim 1 are appropriately described.
- There is an advisory board that includes professionals, parents, and individuals with ASD.

- Letters of support from Philadelphia School District and the mentor's record of successful intervention with this population is encouraging that recruitment and retention will not be an issue.
- They have narrowed the inclusion criteria to make the results easier to interpret.
- There is much more developmental work now going into EMOtion REgulation (EMORE).

Weaknesses

- More could be said about specifically how teachers will be taught the emotion regulation strategies that the app proposes. This is really the key for the app to actually "work."
- The pilot trial seems large. Is such a large study needed to assess feasibility and acceptability?

4. Mentor(s), Co-Mentor(s), Consultant(s), Collaborator(s)

Strengths

- The primary mentor Dr. Mandell is an experienced mentor and expert in the field.
- Additional mentorship has been added in mHealth as requested in prior critiques.

Weaknesses

- None noted by reviewer.

5. Environment and Institutional Commitment to the Candidate

Strengths

- The environment at the University of Pennsylvania (UPenn) and Children's Hospital of Philadelphia (CHOP) is outstanding.

Weaknesses

- None noted by reviewer.

Study Timeline

Strengths

- It is ambitious but probably feasible.

Weaknesses

- None noted by reviewer.

Protections for Human Subjects

Acceptable Risks and Adequate Protections

Data and Safety Monitoring Plan (Applicable for Clinical Trials Only):

Acceptable

Inclusion Plans

- Sex/Gender: Distribution justified scientifically
- Race/Ethnicity: Distribution justified scientifically

- Inclusion/Exclusion Based on Age: Distribution justified scientifically

Vertebrate Animals

Not Applicable (No Vertebrate Animals)

Biohazards

Not Applicable (No Biohazards)

Training in the Responsible Conduct of Research

Acceptable
Comments on Format (Required):

- Acceptable

Comments on Subject Matter (Required):

- Acceptable

Comments on Faculty Participation (Required; not applicable for mid- and senior-career awards):

- Acceptable

Comments on Duration (Required):

- Acceptable

Comments on Frequency (Required):

- Acceptable

Budget and Period of Support

Recommend as Requested

CRITIQUE 3

Candidate: 1
Career Development Plan/Career Goals /Plan to Provide Mentoring: 2
Research Plan: 3
Mentor(s), Co-Mentor(s), Consultant(s), Collaborator(s): 1
Environment Commitment to the Candidate: 1

Overall Impact: This K01 is a resubmission with training goals of receiving additional training in intervention research along with further training in measurement of psychophysiological indices and use. Training in qualitative methods will also be undertaken. The Candidate is support by an exceptional team of mentors and consultants. The Candidate clearly has a path to being an independent researcher. The research plan of this K01 is to develop an app to identify students with autism spectrum disorder (ASD) in the classroom who are at heightened risk to engage in disruptive behaviors based on increase heart rate. The app would then guide teachers / school personnel towards a behavioral intervention approach. However, it is not considered that heart rate may increase for other reasons (not specific to challenging behaviors) and thus an intervention may be implemented that is unneeded or not appropriate. Further, while there is a table in the research strategy of possible behavioral interventions, these are rather vague and it is unclear how these will be chosen for a specific child. These concerns were raised in the previous review, but they are not addressed in sufficient

detail. Nonetheless, the outstanding mentors and Candidate along with potential impact of this application are notable.

Protections for Human Subjects

Acceptable Risks and Adequate Protections

- Minimal risks

Data and Safety Monitoring Plan (Applicable for Clinical Trials Only):

Acceptable

- A DSMP is well thought out.

Inclusion Plans

- Sex/Gender: Distribution justified scientifically
- Race/Ethnicity: Distribution justified scientifically
- Inclusion/Exclusion Based on Age: Distribution justified scientifically
- No human subject concerns

Vertebrate Animals

Not Applicable (No Vertebrate Animals)

Biohazards

Not Applicable (No Biohazards)

Training in the Responsible Conduct of Research

Comments on Format (Required):

Comments on Subject Matter (Required):

Comments on Faculty Participation (Required; not applicable for mid- and senior-career awards):

Comments on Duration (Required):

Comments on Frequency (Required):

Budget and Period of Support

Recommend as Requested

THE FOLLOWING SECTIONS WERE PREPARED BY THE SCIENTIFIC REVIEW OFFICER TO SUMMARIZE THE OUTCOME OF DISCUSSIONS OF THE REVIEW COMMITTEE, OR REVIEWERS' WRITTEN CRITIQUES, ON THE FOLLOWING ISSUES:

PROTECTION OF HUMAN SUBJECTS: ACCEPTABLE

INCLUSION OF WOMEN PLAN: ACCEPTABLE

INCLUSION OF MINORITIES PLAN: ACCEPTABLE

INCLUSION ACROSS THE LIFESPAN PLAN: ACCEPTABLE

COMMITTEE BUDGET RECOMMENDATIONS: The budget was recommended as requested.

Footnotes for 1 K01 MH120509-01A1; PI Name: Nuske, Heather J

NIH has modified its policy regarding the receipt of resubmissions (amended applications). See Guide Notice NOT-OD-14-074 at http://grants.nih.gov/grants/guide/notice-files/NOT-OD-14-074.html. The impact/priority score is calculated after discussion of an application by averaging the overall scores (1-9) given by all voting reviewers on the committee and multiplying by 10. The criterion scores are submitted prior to the meeting by the individual reviewers assigned to an application, and are not discussed specifically at the review meeting or calculated into the overall impact score. Some applications also receive a percentile ranking. For details on the review process, see http://grants.nih.gov/grants/peer_review_process.htm#scoring.

MEETING ROSTER

The roster for this review meeting is displayed as an aggregated roster that includes reviewers from multiple CSR Special Emphasis Panels of the Aggregate roster for the 2020/01 council round.

This roster for CSR is available at:

http://public.era.nih.gov/pubroster/Reports?DOCTYPE=SEP&DESFORMAT=PDF&AGENDA_SEQ_NUM_P=380750

RESPONSE TO REVIEWERS' COMMENTS

We thank the reviewers for their feedback, which gave us the opportunity to address the following concerns:

1. **Additional discussion might have been provided for how behavioral interventions will be chosen for a specific child (Summary, Reviewer 3).**
   - Interventions will be selected by teachers among evidence-based practices that they are trained on by teacher coaches in Dr. Nuske's center. Teacher selection will then be refined using app data, specifically the physiological stress reduction data relating to each strategy used. Strategies associated with the highest physiological stress reduction (heart rate decrease) will show in the app as the "top strategies" for a given period of time (e.g. summary given at the start of each week based on the previous week's data), so to guide teachers to use the strategies that are most effective for a specific child. Development of the top strategies feature of the app will be conducted with findings of exploratory work in Year 1 and stakeholder input.
2. **However, it is possible that heart rate may increase for other reasons (not specific to challenging behaviors) and thus an intervention may be implemented that is unneeded or not appropriate (Summary, Reviewer 3).**
   - We do acknowledge the possibility of false positives (alarm with no oncoming challenging behavior). However, we prioritize sensitivity over specificity as this implementation tool is low cost, low risk (as emotion regulation/behavior management strategies have positive effects) and has the potential for high reward (preventing challenging behaviors). In Year 1, Dr. Nuske will investigate the false positive and false negative (challenging behavior with no alarm) rates and the proportion of these related to movement and other child factors. All analyses will inform heart rate alarm and movement online/offline thresholds in order to optimize sensitivity/specificity, refine app clinical decision support and prevent alarm fatigue. In Year 2, Dr. Nuske will also interview teachers about alarm fatigue and adjust heart rate thresholds accordingly.
3. **It seems like some of the papers proposed do not clearly fit the research plan. However, translating some of the research done here into academic products may be challenging, since so much of it is product development, so it may be reasonable to seek out other avenues for papers (Reviewer 2).**
   - We appreciate the reviewer's attention to this issue. The reviewer is correct in that the goal of working on these additional manuscripts is for Dr. Nuske to maintain her academic productivity and to report on the findings of the research she has been conducting prior to and during the review of this application.
4. **More could be said about specifically how teachers will be taught the emotion regulation strategies that the app proposes. This is really the key for the app to actually "work" (Reviewer 2).**
   - The center in which Dr. Nuske works has a contract with the School District of Philadelphia through which it provides professional development and consultation to district teachers working with students with autism. Teachers will be trained on emotion regulation strategies as part of their ongoing professional development and in-class coaching. Dr. Nuske will assess fidelity on these strategies through direct observation and provide booster in-class consultation as needed.
5. **The pilot trial seems large. Is such a large study needed to assess feasibility and acceptability (Reviewer 2)?**
   - We have now reduced the pilot trial to be 20 instead of 30 educational teams (10 in each treatment group), including 1-2 children with autism, their teacher and teacher aide. We anticipate that around 50% of teachers will have teacher aides willing to participate in the trial and 50% of teams to only involve one child with autism with challenging behaviors, therefore we anticipate the overall sample size to be approximately 60.
